# Enhanced Conductivity of Multilayer Copper–Carbon Nanofilms via Plasma Immersion Deposition

**DOI:** 10.1007/s40820-024-01628-6

**Published:** 2025-02-05

**Authors:** Haotian Weng, Xiwu Zhang, Xuan Liu, Yunhui Tang, Hewei Yuan, Yang Xu, Kun Li, Xiaolu Huang

**Affiliations:** 1 Key Laboratory for Thin Film and Microfabrication of Ministry of Education, Research Institute of Micro/Nano Science and Technology, Shanghai Jiao Tong University, Shanghai, 200240 People’s Republic of China; 2 Jinduo Yuchen Water Environment Engineering Co., Ltd, Shanghai, 200030 People’s Republic of China; 3 Department of Mechanical Engineering College of Engineering, Shanghai Ocean University, Shanghai, 201306 People’s Republic of China; 4 Faculty of Materials and Manufacturing, Beijing University of Technology, Beijing, 100124 People’s Republic of China

**Keywords:** Copper–carbon nanofilms, Plasma immersion, Carbon layer deposition, Electron mobility

## Abstract

Although room-temperature superconductivity is still difficult to achieve, researching materials with electrical conductivity significantly higher than that of copper will be of great importance in improving energy efficiency, reducing costs, lightening equipment weight, and enhancing overall performance. Herein, this study presents a novel copper–carbon nanofilm composite with enhanced conductivity which has great applications in the electronic devices and electrical equipment. Multilayer copper–carbon nanofilms and interfaces with superior electronic structures are formed based on copper materials using plasma immersion nanocarbon layer deposition technology, effectively enhancing conductivity. Experimental results show that for a five-layer copper–carbon nanofilm composite, the conductivity improves significantly when the thickness of the carbon nanofilm increases. When the carbon nanofilm accounts for 16% of the total thickness, the overall conductivity increases up to 30.20% compared to pure copper. The mechanism of the enhanced conductivity is analyzed including roles of copper atom adsorption sites and electron migration pathways by applying effective medium theory, first-principles calculations and density of states analysis. Under an applied electric field, the high-density electrons in the copper film can migrate into the nanocarbon film, forming highly efficient electron transport channels, which significantly enhance the material’s conductivity. Finally, large-area electrode coating equipment is developed based on this study, providing the novel and robust strategy to enhance the conductivity of copper materials, which enables industrial application of copper–carbon nanocomposite films in the field of high conductivity materials.

## Highlights

- With plasma immersion deposition technology, multilayer copper–carbon nanofilms were fabricated and conductivity can achieve up to 30.20% increase compared to pure copper.
- By applying effective medium theory, first-principles calculations, and density of states analysis, the critical roles of copper atom adsorption sites and electron migration pathways within the nanocarbon film were analyzed, elucidating the mechanism of the conductivity enhancement.
- Large-scale electrode coating equipment suitable for industrial production was developed.

## Graphical abstract

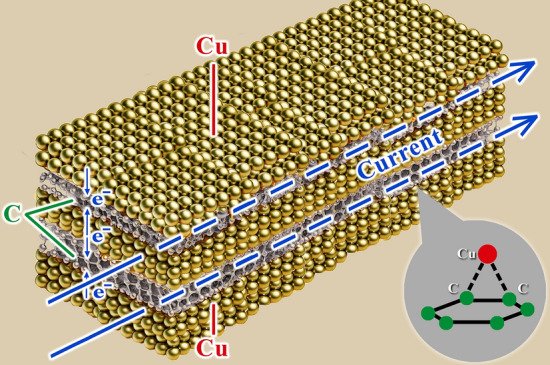

## Introduction

In recent years, more and more high-performance conductive materials have been extensively investigated owing to the rapid development of electronic devices and electrical equipment [1]. Ideally, superconductors would achieve the highest conductivity. However, since room-temperature superconductivity remains unattainable, copper, as the second most conductive metal after silver, has become widely used in practical applications due to its lower cost and abundant availability [2]. Enhancing the conductivity of copper holds significant importance in improving energy efficiency, reducing costs, decreasing equipment weight, and enhancing overall performance. Consequently, this has become a key research focus in the fields of physics and materials science [3]. Copper can provide exceptional conductivity with the property of high electron density of approximately 1.9 × 10^29^ electrons m^−3^ [3, 4]. However, significant electron–electron scattering in copper results in relatively low electron mobility, about 43 cm^2^ V^−1^ s^−1^, which limits the potential enhancements in conductivity [5, 6]. To overcome this challenge, an innovative approach combining copper with carbon-based nanocomposites has been extensively studied [7]. These composites not only retain the high electron density characteristics of copper but also enhance its conductivity by introducing carbon materials, fully utilizing the unique structural advantages of carbon. They exhibit excellent electrical, mechanical, and chemical properties [8, 9], thereby showing great potential for applications in the field of conductive materials [10].

Nanocarbon thin films, such as carbon nanotubes and graphene can present great potential for future applications in high-conductivity materials owing to their ultra-high electron mobility, which exceed 10,000 cm^2^ V^−1^ s^−1^ [11–13]. Therefore, a strategy of combining copper and nanocarbon film was proposed, in order to enhance the composite’s electrical conductivity significantly and address copper’s mobility limitation [14–16]. Numerous developments have been reported on the copper and nanocarbon film combination. In 2012, Kasichainula et al. achieved a 15–17% increase in conductivity when they dispersed graphene oxide into a copper matrix using electrochemical deposition to produce a composite with high conductivity and low resistance [17, 18]. In 2014, Xie et al. developed a one-step electrochemical process to synthesize a composite film with excellent electrical contact properties by co-deposited reduced graphene oxide (rGO) and copper. This study showed that the composite’s resistivity was lower than polished copper foil and electroplated copper films, which had 12% increase of conductivity [19]. In 2016, Huang et al. reported a composite film with embedding multilayer graphene into a copper matrix that has enhanced mechanical and electrical properties. The film’s resistivity was 1.66 × 10^−8^ Ω m, around 5% lower than pure copper films [20]. In the same year, Dong et al. demonstrated that the composite has high conductivity (8% increase) and exhibits high hardness by incorporating graphene into W_70_Cu_30_ composites using mechanical alloying and pressureless infiltration sintering [21]. In 2017, Luo et al. produced a composite with excellent conductivity and mechanical properties by dispersing a silver/reduced graphene oxide (Ag-RGO) mixture into a copper matrix through ball milling and hot-press sintering at 800 °C. Under 50 MPa pressure, the conductivity improved by approximately 18.6% [22]. Therefore, it is expected that the incorporation of carbon nanofilms into copper materials can significantly enhance their conductivity.

In our previous research, Liu et al. successfully prepared CNTs/Cu composite films using a combination of electrophoresis and electroplating methods [23]. This approach, carbon nanotubes dispersing uniformly within the copper matrix, can enhance electrical performance and reduce resistance by over 20%. This improvement of performance is mainly attributed to the increased grain size in the composite films and the preferential orientation of the Cu (111) crystal plane. The results showed that the CNTs/Cu composite materials exhibited superior electrical properties compared to traditional pure copper films, presenting the possibility for further improvement of interconnect materials in nanoelectronics. However, the specific mechanism of the enhanced conductivity was not thoroughly analyzed. Based on the previous work, we present that the electrical conductivity of multilayer copper–carbon nanofilms can be further optimized by plasma immersion deposition technology. The relationship between the conductivity of the composite material and the thickness of the copper–carbon films was fully investigated. Furthermore, the mechanism of the enhanced conductivity was analyzed including roles of copper atom adsorption sites and electron migration pathways by applying effective medium theory, first-principles calculations and density of states analysis.

Unlike the traditional thin-film deposition methods, such as electrophoresis and electroplating, with the limitation of carbon deposition efficiency, interface quality and scalability for industrial applications [24–26], plasma-based thin film deposition technology offers a more efficient and controllable solution to create uniform and high-quality nano-thin film [27–30]. In 2022, Cech et al. presented that plasma chemistry enables precise control over thin-film properties by adjusting the balance of carbon and silicon species, which directly influences film composition and structure [31]. In 2024, Li et al. reported that the plasma can enhance the adhesion of GLC films, which improves mechanical properties, enhances wear resistance, and effectively reduces the friction coefficient, thereby extends the material's service life [32]. The same year, Wang et al. demonstrated that high-quality films produced by plasma under lower ablation energies, can offer insights for optimizing the deposition process [33]. Otaka et al. achieved high-precision control in film deposition by regulating the physical properties of the films through the control of capacitively coupled plasma [34]. However, how to implement plasma technology in large-scale industrial production still remains a significant challenge [35–37].

This paper reports a facile and robust approach to create uniform copper–carbon nanofilms using plasma immersion technology. The conductivity of the copper–carbon nanofilms was tested and showed an enhancement of up to 30.20% compared to copper nanofilms of the same thickness. In order to fully understand the specific mechanisms of the conductivity improvement, effective medium theory, first-principles calculations, and density of states analysis were applied. Large-area electrode coating equipment was developed based on this novel approach, which enables industrial application of copper–carbon nanocomposite films in improving copper conductivity and in the field of high conductivity materials.

## Experimental Section

### Plasma Immersion Carbon Layer Deposition Technology

The copper–carbon multilayer nanofilm composite material in this study was deposited using a carbon layer deposition technique under plasma immersion conditions. The principle of the carbon layer deposition method involves introducing gaseous raw materials into a reaction chamber, where these gases interact under suitable temperature and pressure conditions, leading to the formation of new solid-phase compounds through a series of chemical reactions [38–40]. These newly formed compounds subsequently deposit onto the surface of the substrate placed in the reaction chamber, forming a uniform thin film [41]. A major advantage of the plasma immersion deposition technique is its ability to achieve uniform carbon layer deposition over large substrate areas, a feature that supports large-scale equipment development and is particularly crucial for mass production. Moreover, since plasma immersion deposition can be carried out at relatively low temperatures, it is suitable for materials and substrates that cannot withstand high-temperature treatments. By regulating the introduced plasma, the deposition process can be optimized under various conditions, enabling precise control over the carbon layer’s properties, including enhanced uniformity, increased material purity, and adjustable microstructure. Introducing plasma into the carbon layer deposition process provides a large number of active particles in the deposition chamber, significantly improving the uniformity and compactness of the copper surface nano-carbon film [42, 43]. The microscopic mechanism that alters the material's properties involves several aspects:

1. Charge injection and surface polarization: Plasma immersion conditions can inject charge into the material surface or cause surface polarization, particularly in dielectric and semiconductor materials. This charge injection can alter the material’s resistivity and dielectric properties. For resistivity $\rho$, the following applies:
  1 $$\rho = \frac{1}{\sigma } = \frac{1}{ne\mu }$$
  where $\sigma$ is the electrical conductivity, $n$ is the carrier concentration, $e$ is the electron charge, and $\mu$ is the mobility. Under plasma immersion conditions, the injection of doping atoms introduces high-energy electrons or ions that generate additional electrons or holes at the material’s surface, thereby increasing the carrier concentration $n$ in the surface or near-surface region. Plasma immersion can also repair lattice defects by reducing defects and inhomogeneities in the material, which decreases scattering effects and thus improves carrier mobility $\mu$:
  2 $$\mu = \frac{e\tau }{{m^{*} }}$$
  where $\tau$ is the average free time of carriers, and $m^{*}$ is the effective mass of the carriers. Plasma reduces the defect density, increasing the average free time $\tau$ of carriers, thereby improving their mobility $\mu$, which ultimately affects the resistivity $\rho$.
2. Surface morphology modification: Plasma immersion alters the surface morphology of the material through physical sputtering. When high-energy particles bombard the material’s surface, part of the material can be removed, forming micro- or nanoscale surface structures. For this process, there are:
  3 $$Y = \frac{{N_{i} E_{i} }}{{U_{s} }}$$
  where $Y$ is the sputtering yield, $N_{i}$ is the number of incident ions, $E_{i}$ is the energy of the incident ions, and $U_{s}$ is the surface binding energy. The number of ions impacting the material surface is determined by the plasma density and ionization rate. The higher the plasma density and the higher the ionization rate, the larger the number of incident ions $N_{i}$, which is also influenced by the plasma generation conditions, such as power, gas type, and pressure. At the same time, the electric field and energy in the plasma determine the acceleration of the ions, which affects the energy of the incident ions $E_{i}$. For example, higher RF power can increase the ion acceleration energy, thereby increasing $E_{i}$.
3. Plasma immersion conditions can introduce various functional groups that significantly modify the material’s surface energy. These functional groups may increase or decrease surface energy, influencing the material’s wettability, adhesion, and biocompatibility.
4. Repair of Internal Stress and Defects: Plasma immersion conditions can modify the material’s microstructure, relieving internal stress and eliminating defects.

The carbon layer deposition in this study was primarily carried out in the negative glow region on the surface of the RF electrode, which has high-density plasma. This choice is based on the negative glow region’s high plasma density, which provides a large number of high-energy electrons and ions to promote chemical reactions, enhance deposition rates, and improve film quality. This allows for efficient deposition in a short time, enhancing process efficiency. Moreover, the plasma density in the negative glow region is relatively uniform, enabling the formation of consistent film thickness on the substrate surface, which is crucial for many high-precision applications.

Of course, excessively high plasma density during carbon layer deposition under plasma immersion conditions may also lead to some negative effects, such as uneven deposition rates affecting the overall performance of the interface layer or incomplete gas-phase reactions due to excessive temperature caused by high plasma density. In practical tests, it was found that when the equipment power was controlled at 600 W and the temperature was 150 °C, spectral tests showed a plasma density of 10^12^ cm^−3^ on the electrode surface, and the nanocarbon film and interface layer produced under these conditions exhibited good performance.

### Preparation of the Copper–Carbon Composite Nanofilms

**Fig. 1:**
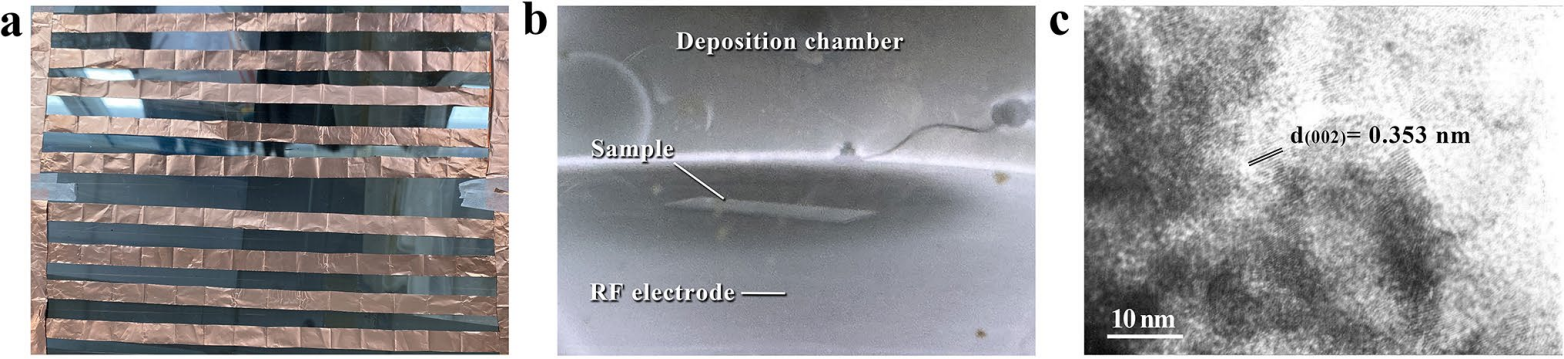
**a** Copper foil for sample preparation. **b** Operating state of the carbon layer deposition chamber. **c** High-resolution TEM image of nanocarbon film

To further investigate the effects of nanocarbon films on copper materials and the conductivity of multilayer copper–carbon nanofilm composite, three different types of copper–based materials were selected for testing in this study. These materials include: 6 µm thick double-sided polished copper foil (as shown in Fig. 1a), 23 µm diameter copper wire, and copper thin films with controlled thickness prepared by magnetron sputtering. The reason for conducting surface carbon layer material tests based on copper thin films is that the parameters during magnetron sputtering allow for control over the thickness of the copper film. This enables an investigation of the conductivity changes when both the copper layer and the carbon film layer are thin, as well as the specific changes when the copper layer thickness increases. This further helps explore the bonding state of the copper–carbon interface layer. Additionally, multilayer copper–carbon nanofilms can be prepared by alternating the growth of copper thin films and carbon layers.

During the sample preparation stage, it is first necessary to record the initial resistance values of the three materials. To facilitate precise resistance measurements later using a micro-ohmmeter, the ends of the samples need to be masked before depositing the nanocarbon film. The samples are then placed in the deposition equipment. Figure 1b shows the operating state of the carbon layer deposition chamber. The sample is placed on the surface of a 50 cm diameter RF electrode, where nanocarbon films are deposited under plasma immersion conditions within the negative glow region. The deposition process is conducted at a controlled power of 600 W and a temperature of 150 °C, with spectral analysis yielding a plasma density of 10^12^ cm^−3^. By adjusting the deposition time, the thickness of the nanocarbon film can be controlled. Considering that the first two types of samples are relatively fragile, the vacuum state of the carbon deposition equipment must be removed carefully and slowly after preparation to prevent airflow damage to the samples. Figure 1c presents the high-resolution TEM image of the prepared nanocarbon film, revealing distinct lattice fringes. The lattice spacing of 0.353 nm corresponds to the (002) crystal plane, exhibiting nanoscale ordering.

After removing the samples, their resistance values are measured using a micro-ohmmeter. Notably, for the copper foil samples, after the growth of the nanocarbon film on one side, the samples need to be flipped for a second deposition to test the impact of double-sided nanocarbon film growth on the material's conductivity. Based on this, a high-temperature annealing experiment is introduced to further explore the effect of the nanocarbon film hybridization on the conductivity of the copper–carbon interface layer.

## Results and Discussion

### Experimental Results

#### Conductivity of Copper–Carbon Composite Nanofilms Based on Copper Foil

**Fig. 2:**
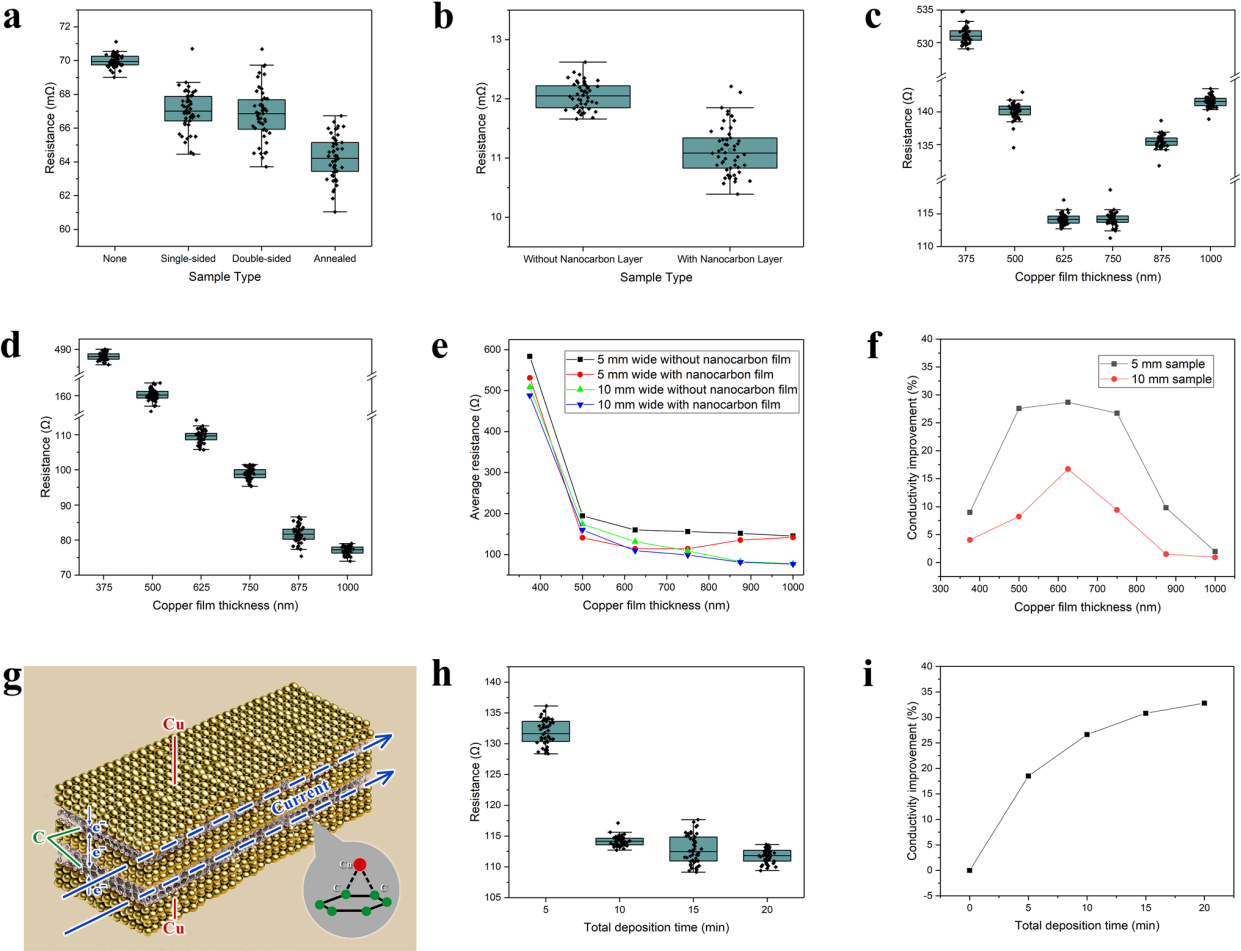
**a** Box plot for the resistance of copper foil samples. **b** Box plot for the resistance of copper wire samples. **c** Box plot of 5 mm wide sample resistance after carbon layer growth. **d** Box plot of 10 mm wide sample resistance after carbon layer growth. **e** Comparison of average sample resistance before and after carbon layer growth. **f** The conductivity improvement effect of nanocarbon films on copper films. **g** Schematic diagram of copper–carbon composite film. **h** Box plot of sample resistance after carbon layer deposition for different total durations. **i** The relationship between conductivity improvement and total deposition time

A total of 50 sample sets were prepared for testing in this study. The test results are shown in the box plot in Fig. 2a. After calculations, the following average resistance values were obtained: the resistance of the copper foil sample without a carbon layer on the surface was 69.76 mΩ, the resistance of the copper foil sample with a single-sided nano-carbon film was 66.74 mΩ, and the resistance of the copper foil sample with a double-sided nano-carbon film was 66.60 mΩ. Furthermore, the double-sided nano-carbon film copper foil sample, after high-temperature annealing, exhibited an even lower resistance of 64.18 mΩ.

The experimental results showed that the nanocarbon film increased the conductivity of the original copper foil by 4.3%, while the double-sided nanocarbon film improved the conductivity by 4.5%, slightly higher than the effect of the single-sided film. To further enhance the effect of the nanocarbon film on the conductivity of the copper foil, a high-temperature annealing experiment was conducted. The annealing process was carried out in a tube furnace under nitrogen protection to prevent the oxidation of the copper material. Considering the high heat resistance of the copper foil, the annealing process was set at 700 °C. After annealing, the conductivity of the double-sided nanocarbon film copper foil samples achieved a significant increase of 7.99%. As shown in the box plot in Fig. 2a, the experimental data exhibited good consistency.

For the phenomenon that the conductivity of the samples with single-sided and double-sided carbon layer deposition was similar, analysis suggests that this is because there was no significant difference in the hybridization of the double-sided and single-sided nanocarbon films, and the number of electrons that copper atoms could transfer to carbon atoms did not change much, resulting in only a slight improvement in conductivity. However, after high-temperature annealing, the carbon atoms bonded more tightly with the copper atoms on the copper foil surface, enhancing the interaction between the atoms. Additionally, the increased orderliness of the nanocarbon film, with the hexagonal-like structure becoming more prominent, further improved the conductivity [23, 44].

#### Conductivity of Copper–Carbon Composite Materials Based on Copper Wire

To further verify the impact of the nano-carbon film on the conductivity of copper materials, this study selected 50 sets of copper wires with a diameter of 23 µm and a length of 24 cm as samples. Following the same pre-treatment used for the copper foil samples, a 10-min nanocarbon film deposition was carried out under conditions of 600 W power, 150 °C, and high-density plasma immersion on the RF electrode surface. The experimental results are shown in Fig. 2b.

The average resistance of the initial samples was calculated to be 12.06 Ω, and after the growth of the nanocarbon film, the average resistance decreased to 11.13 Ω. As shown in the box plot in Fig. 2b, the experimental data exhibited good consistency. Based on the above data, it can be concluded that the conductivity of the 23 µm diameter copper wire treated with the nanocarbon film increased by 7.71%. This result clearly demonstrates the effectiveness of the nanocarbon film in improving the conductivity of copper wire materials.

#### Conductivity of Single-Layer Copper–Carbon Composites Nanofilms Based on Copper Films with Different Thicknesses

Given that the thickness of the copper layers in copper foil and copper wire cannot be adjusted, copper films of different thicknesses and widths need to be prepared in advance to test the effect of nanocarbon film growth on thinner copper materials. In the specific experimental process, copper film samples were prepared using magnetron sputtering. After preparation, the glass plates with copper films were placed in the carbon layer deposition equipment, and the ends of the copper films were covered to avoid being affected by the deposition. Methane gas was used as the carbon source for carbon layer deposition, with the deposition temperature controlled at 150 °C. Under plasma immersion conditions with a power of 600 W, deposition was carried out for 10 min, resulting in a nanocarbon film with a thickness of 50 nm on the surface of the copper film. The conductivity of the copper–carbon composite nanofilms was then measured. Finally, the collected data were compiled to determine under which set of parameters the copper–carbon composite nanofilms achieved optimal conductivity.

In the experiment, 5 mm wide copper films and 10 mm wide copper films were prepared for testing. The step profiler measurements indicated that for every additional 5 min of sputtering, the thickness of the copper film increased by approximately 125 nm. The sputtered thickness was subsequently used to distinguish and describe different copper film samples. For the 5 and 10 mm wide copper film samples, 50 sets were selected for each thickness of 375, 500, 625, 750, 875, and 1000 nm. After 10 min of nanocarbon film growth in the carbon layer deposition equipment, the test data are shown in Fig. 2c–e.

It is noteworthy that during the treatment of the 5 mm wide samples, although the nanocarbon film was grown for the same 10 min, the resistance of the sample based on the 750 nm thick copper film was smaller than that of the 875 nm thick sample as shown in Fig. 2e. This phenomenon is related to the quality of the interface layer and contact resistance. Since the copper films were prepared by magnetron sputtering, variations in the growth process can affect the contact quality of the interface layer between the copper and the nanocarbon film. A thinner copper film (750 nm) can form better interfacial adhesion and create a network structure of electron transport channels, resulting in lower interface resistance. In contrast, a thicker copper film (875 nm) will have poor interfacial contact with the nanocarbon film due to stress and interface mismatch, which in turn increases the interface layer resistance.

Based on the above data, the effect of the nanocarbon film on the improvement of the conductivity of copper–carbon materials with different copper film thicknesses after 10 min of carbon layer deposition can be plotted, as shown in Fig. 2f. For the 5 mm wide samples, the nanocarbon film formed through carbon layer deposition under plasma immersion conditions significantly improved the conductivity of the resulting copper–carbon material. When the deposition time of the nanocarbon film was fixed at 10 min, the effect of the nanocarbon film (corresponding to a thickness of 50 nm) on improving conductivity initially increased and then decreased as the copper layer thickness increased. When the copper layer sputtering time was 25 min, with a thickness of 625 nm, the conductivity enhancement reached its maximum, with an increase of 28.61%. Similarly, for the 10 mm wide samples, after 10 min of nanocarbon film deposition, the conductivity improvement also showed a trend of first increasing and then decreasing with the increase in copper layer sputtering time and thickness. Likewise, when the copper layer sputtering time was 25 min, with a thickness of 625 nm, the conductivity enhancement reached its peak, with an increase of 17.14%.

From a theoretical perspective, when an ordered nanocarbon film grows on a copper film, the copper film provides a large number of transferable electrons, and the distribution of copper and carbon atoms in the composite nanofilm material forms a network structure. Due to the quasi-one-dimensional structure of the nanocarbon film and the staggered arrangement of the charge densities in its highest occupied molecular orbital (HOMO) and lowest unoccupied molecular orbital (LUMO), channels that are more conducive to electron transport are formed. A significant number of electrons from the copper film in contact with the nanocarbon film migrate to it, ultimately leading to an improvement in the conductivity of the copper–carbon composite material. In the initial stages, as the copper film thickness increases, surface roughness decreases, allowing the nanocarbon film to cover the copper surface more uniformly. This promotes the formation of a network structure and reduces scattering during electron transport, thereby enhancing conductivity. However, when the copper film reaches a certain thickness (around 625 nm), further increases in copper thickness result in diminishing marginal benefits from the additional electron transport channels provided by the nanocarbon film, leading to a decrease in the rate of improvement. Additionally, the internal structural complexity of the film increases, and an excessively thick copper film can lead to increased stress within the interface layer, affecting the structure and conductivity of the nanocarbon film in contact with it. Therefore, the conductivity enhancement effect of the nanocarbon film begins to weaken after the copper film reaches a certain thickness. For copper films with a thickness of 625 nm, the growth of the nanocarbon film reaches an optimal balance, resulting in the most significant improvement in conductivity.

#### Conductivity of Multilayer Copper–Carbon Composite Nanofilms Based on Nano-Carbon Films with Different Thicknesses

To investigate the effect of the nanocarbon film thickness on the conductivity of multilayer copper–carbon materials using carbon layer deposition technology under plasma immersion conditions, the 625 nm thick, 5 mm wide copper film sample—where the nanocarbon film showed the most significant conductivity improvement—was used as the baseline. Multilayer copper–carbon nanofilms films of the same thickness and width were prepared. The composite films were alternately grown by magnetron sputtering for the copper films and plasma immersion carbon layer deposition for the nanocarbon films, forming a multilayer structure with three layers of copper films and two layers of carbon films as shown in Fig. 2g. Nanocarbon films were grown in different batches for total times of 5, 10, 15, and 20 min, corresponding to thicknesses of 25, 50, 75, and 100 nm, respectively, and resistance were measured. The results are shown in Fig. 2h, i.

As shown in Fig. 2i, with the increase in total carbon layer deposition time under plasma immersion conditions, the overall conductivity gradually improves. However, the rate of conductivity improvement decreases as the thickness of the nanocarbon film increases, generally exhibiting a logarithmic trend. This phenomenon is also validated in the effective medium analysis section later as well. For the multilayer copper–carbon film samples with a total thickness of 625 nm and a width of 5 mm, when the total carbon layer deposition time is 20 min (corresponding to three layers of 175 nm copper film and two layers of 50 nm carbon film, with the carbon film accounting for 16% of the total thickness), the resistance is reduced by 30.20% compared to a pure copper film of the same thickness.

The underlying mechanism behind this change in the magnitude of improvement can be explained by the principle of how nanocarbon films enhance the conductivity of copper materials. When carbon atoms in the nanocarbon film interact with surface copper atoms, they form a highly ordered network structure, providing smoother pathways for electron transport. As the thickness of the nanocarbon film increases, this highly ordered network structure expands, thereby enhancing electron mobility. In addition, a thicker nanocarbon film can more effectively cover surface irregularities of the copper substrate, reducing scattering during electron transport and further improving conductivity. However, once the nanocarbon film reaches a certain thickness, its marginal effect on conductivity improvement begins to decrease. Additionally, increasing the thickness of the carbon layer also reduces the copper content per unit volume. According to the research previously conducted by our group [23], the copper–carbon ratio affects the overall material’s grain size and crystal plane peak size. Furthermore, as the improvement in conductivity is based on a comparison with pure copper films of the same thickness, the conductive properties of the composite material are influenced not only by the high electron mobility of the carbon film but also by the contribution of electrons from the copper film. Therefore, the thickness of the carbon film needs to be optimized to find the best balance between improving conductivity and maintaining sufficient copper content in practical applications. Guided by theoretical analysis and validated by experiments, when the total deposition time of the carbon film is 20 min, including two layers of 50 nm thick carbon films, the 625 nm thick multilayer copper–carbon composite nanofilms exhibits the most significant improvement in conductivity, achieving the best enhancement compared to pure copper material, with an improvement rate of 30.20%.

### Theoretical Analysis and Discussion

The above experimental results indicate that as the thickness of the nanocarbon film increases, the conductivity of the multilayer copper–carbon composite material significantly improves, with a maximum enhancement of 30.20%. Utilizing effective medium theory, first-principles calculations, and density of states analysis, this paper investigates the adsorption behavior of copper atoms in the nanocarbon film and its impact on electron migration pathways, revealing the underlying reasons for this phenomenon.

#### Effective Medium Analysis of Electrical Resistivity Changes

The effective medium theory (EMT) is a theoretical framework used to describe the physical properties of different components in composite materials. It has broad applications in the study of composite materials and can be used to describe the impact of nanoscale metals on the overall performance of composite film materials [45, 46]. EMT assumes that the conductivities of copper and carbon materials form an equivalent effective medium within the composite material, and the overall conductivity of the composite can be calculated based on the volume fractions and conductivities of both materials [47].

Assume that the grain size of the composite material is small compared to the sample size, so the grains can be considered isotropically distributed. In this way, the electrical conductivity of the composite material is also isotropic. For a composite material composed of substance A and substance B, suppose the conductivity of substance A is $\sigma_{1}$ and its volume fraction is $f_{1}$, while the conductivity of substance B is $\sigma_{2}$ and its volume fraction is $f_{2}$, with the relationship $f_{1} + f_{2} = 1$. Assuming that the spatial current through the sample is uniform, the range of the effective conductivity of the composite material can be derived as:

4 $$\left( {\frac{{f_{1} }}{{\sigma_{1} }} + \frac{{f_{2} }}{{\sigma_{2} }}} \right)^{ - 1} \le \sigma \le f_{1} \sigma_{1} + f_{2} \sigma_{2} .$$

This represents the absolute boundary condition of the composite material, known as the Wiener boundary condition. 

When the composite material is composed of N types of materials, the range of its effective conductivity is:

5 $$\left( {\mathop \sum \limits_{i = 1}^{N} \frac{{f_{i} }}{{\sigma_{i} }}} \right)^{ - 1} \le \sigma \le \mathop \sum \limits_{i = 1}^{N} f_{i} \sigma_{i} .$$

When $f_{2}$ < < 1, meaning that material B is in a highly diluted suspension state, it is discontinuous in the composite material. Its shape can be assumed to be ellipsoidal, and at this time, the following applies:

6 $$\sigma_{{{\text{eq}}}} = \sigma_{1} + f_{2} \frac{{\left( {\sigma_{2} - \sigma_{1} } \right)}}{3}\mathop \sum \limits_{i = 1}^{3} \frac{{\sigma_{1} }}{{\sigma_{1} + A_{i} \left( {\sigma_{2} - \sigma_{1} } \right)}}$$

where $\sigma_{{{\text{eq}}}}$ is the effective conductivity, and *A* is the depolarization factor along the ellipsoid axis, as shown in Table 1.

**Table 1:** Depolarization factor

	$A_{1}$	$A_{2}$	$A_{3}$
Sphere	1/3	1/3	1/3
Oblate ellipsoid	1	0	0
Prolate ellipsoid	0	1/2	1/2

Thus, the effective conductivity of a composite material composed of randomly distributed substances in a diluted suspension state can be obtained as:

7 $$\sigma_{{{\text{eq}}}} = \sigma_{1} + f_{2} \frac{{\left( {\sigma_{2} - \sigma_{1} } \right)\left( {5\sigma_{1} + \sigma_{2} } \right)}}{{3\left( {\sigma_{1} + \sigma_{2} } \right)}}.$$

When $f_{2}$ does not satisfy $f_{2}$ < < 1, a similar method can still be used for the calculation. However, it is necessary to assume that material B is coated by a substance with an effective conductivity of $\sigma^{*}$ rather than by a substance with a conductivity of $\sigma_{1}$. $\sigma^{*}$ is merely an approximation of the true effective conductivity of the composite material, leading to:

8 $$\sigma^{*} = \sigma_{1} + f_{2} \frac{{\left( {\sigma_{2} - \sigma_{1} } \right)\sigma^{*} }}{{2\sigma^{*} + \sigma_{2} }}.$$

Thus, it can be obtained that:

9 $$\frac{{\sigma^{*} - \sigma_{1} }}{{2\sigma^{*} + \sigma_{1} }}f_{1} + \frac{{\sigma^{*} - \sigma_{2} }}{{2\sigma^{*} + \sigma_{2} }}f_{2} = 0.$$

For the composite material composed of nanocarbon film and copper, by substituting the resistivity of copper at room temperature $\rho_{{{\text{Cu}}}} = 1.67\;\upmu \Omega \;{\text{cm}}$ and the resistivity of the carbon film $\rho_{{\text{C}}} = 0.35\;\upmu \Omega \;{\text{cm}}$ into the equation, the following result is obtained:

10 $$\frac{{\frac{1}{{\rho^{*} }} - \frac{1}{{\rho_{Cu} }}}}{{2\frac{1}{{\rho^{*} }} + \frac{1}{{\rho_{Cu} }}}}f_{1} + \frac{{\frac{1}{{\rho^{*} }} - \frac{1}{{\rho_{C} }}}}{{2\frac{1}{{\rho^{*} }} + \frac{1}{{\rho_{C} }}}}f_{2} = 0.$$

That is:

11 $$\rho^{*} = 0.485 - 1.98f_{2} + \frac{1}{2}\sqrt {\left( {0.97 - 3.96f_{2} } \right)^{2} + 4.676} .$$

**Fig. 3:**
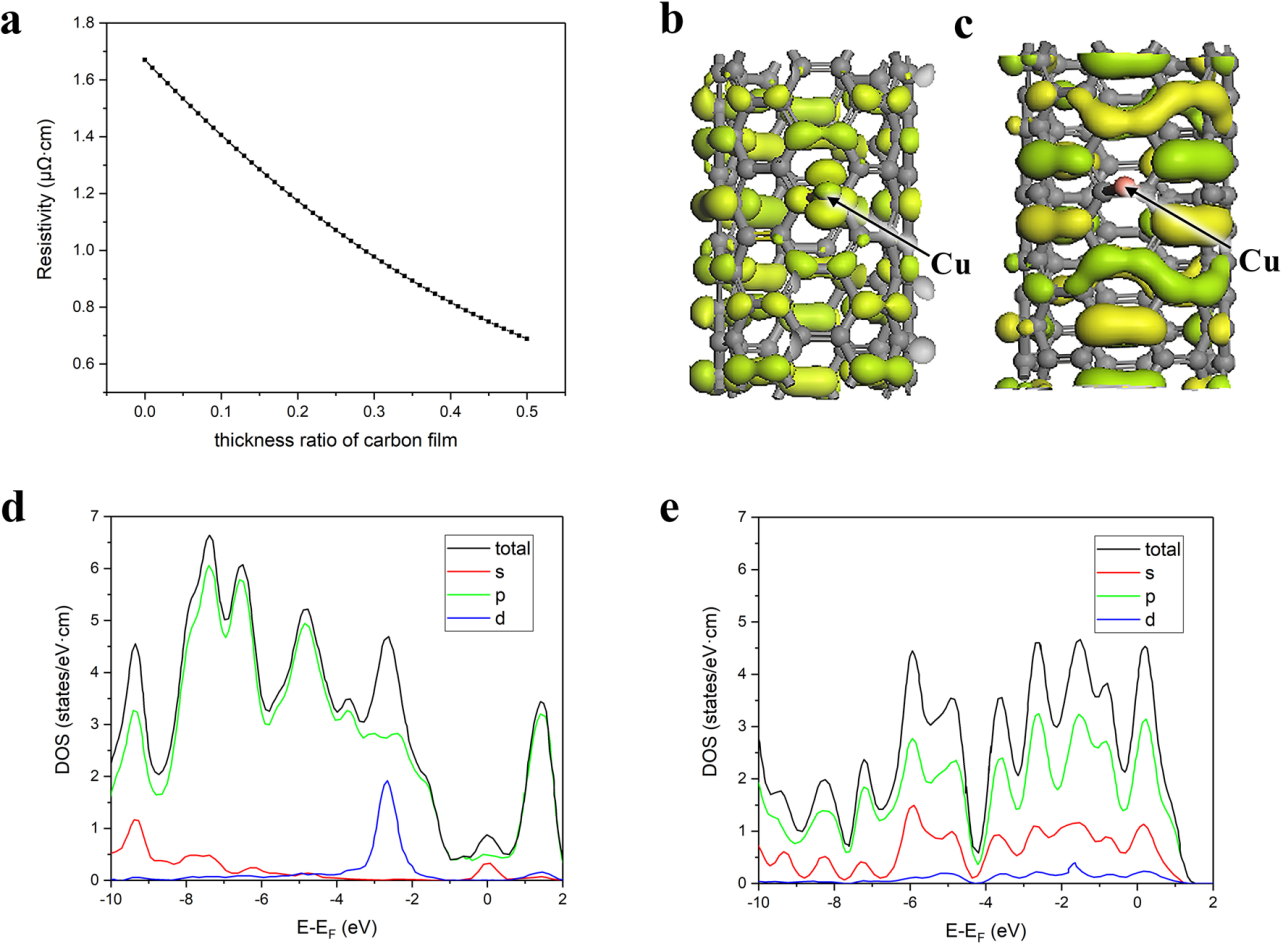
**a** Effect of nanocarbon film proportion on the conductivity of copper–carbon composite materials. **b** HOMO and LUMO charge density distribution with copper atom adsorbed at the bridge site without applied electric field. **c** HOMO and LUMO charge density distribution with copper atom adsorbed at the bridge site with applied electric field. **d** DOS of the copper–carbon composite film material system without applied electric field. **e** DOS of the copper–carbon composite film material system with applied electric field

The plot is obtained as Fig. 3a. It can thus be concluded that the resistivity of the copper–carbon composite film material decreases as the proportion of the nano-carbon film in the composite material increases. Additionally, as the thickness of the carbon film increases, the marginal effect on conductivity enhancement correspondingly diminishes, aligning with the experimental results presented in this study.

#### First-Principles Calculation of Electron Behavior

First-principles calculation, based on density functional theory (DFT), is a commonly used method for studying the microscopic electronic structure of materials [48]. In the fields of condensed matter physics and materials science, first-principles calculation methods rely on DFT, utilizing fundamental approximations within physical models and solving through mathematical methods such as variational or perturbation methods, without relying on any empirical or semi-empirical parameters. Its core lies in rigorously solving the Schrödinger equation to facilitate the study of material properties and the computational design of new materials [49].

To investigate the changes in conductivity of copper and nano-carbon film composites, the model can be simplified by first analyzing the effect on electron behavior when a single copper atom is combined with the nano-carbon film structure. The conductivity of a conductor is formed by the directional movement of electrons under the influence of an external field. Copper, as a good conductor, has a diffuse distribution of electronic wave functions within it. When copper material is combined with a nano-carbon film, it affects the distribution of local electrons, thereby impacting the overall conductivity on a macroscopic scale [50]. Using first-principles calculation methods, the electron distribution of the copper and nano-carbon film composite can be compared under conditions with and without an applied electric field, thus providing an analysis of the experimental results.

The computational model uses the structure of a single copper atom adsorbed on a nano-carbon film. Since the carbon atoms on the carbon layer exist in a quasi-hexagonal form, the copper atom can theoretically adsorb at multiple positions [14]. Pristine nano-carbon film provides three inequivalent sites for metal adsorption: the vacancy site (above the C_6_ ring), the bridge site (above the C–C bond), and the top site (above a C atom). For the calculations of bonding structure and bonding energy, a pseudopotential plane-wave basis set function is used, with exchange–correlation treated by the generalized gradient approximation method. Spin-polarized and non-spin-polarized calculations are performed for quasi-hexagonal carbon atoms without and with copper atom adsorption, respectively. The most probable adsorption site can be determined by comparing bonding energies:

12 $$E_{b} = E_{{T\left( {\text{C}} \right)}} + E_{{T\left( {{\text{Cu}}} \right)}} - E_{{T \left( {{\text{Cu}} + {\text{C}}} \right) }}$$

where $E_{{T\left( {\text{C}} \right)}}$ represents the total energy of the quasi-hexagonal carbon atoms, $E_{{T\left( {{\text{Cu}}} \right)}}$ represents the total energy of a single copper atom, and $E_{{T{ }\left( {{\text{Cu}} + {\text{C}}} \right){ }}}$ represents the total energy of the quasi-hexagonal carbon atom system with copper atom adsorption.

For the case where the copper atom is initially positioned directly above the center of the quasi-hexagonal carbon atoms (the vacancy site), after structural relaxation and optimization, its final adsorption position is close to the bridge site, i.e., above the midpoint of the carbon–carbon bond. At the same time, the calculated bonding energy difference is approximately 0.70 eV, indicating that the adsorption structure of the copper atom at the bridge site is more stable than at the top site. Therefore, in copper–carbon composites, the preferred adsorption site for the copper atom is the bridge site, located above the midpoint of the carbon–carbon bond, where it bonds with two carbon atoms, with a bond length of approximately 2.1–2.2 Å and a formation energy of around 0.69 eV.

On this basis, Mulliken population and differential charge density are commonly used to analyze the charge transfer within the system. In the calculation process, the applied external electric field is directed along the axis of the quasi-hexagonal structure of the carbon atoms, with a strength of 0.5 eV Å^−1^, and it is assumed that the electric field intensity is uniformly distributed. Mulliken population analysis of the copper–carbon composite interfacial layer system shows that the copper atom carries a positive charge, while the connected carbon atoms carry a certain amount of negative charge. Additionally, the differential charge density distribution results also indicate that in the copper–carbon composite interfacial layer material system, charge migrates from the copper atom to the carbon atoms, facilitating electron transfer. Under an applied external electric field, such transferred charge increases several times, this phenomenon also reflected in the analysis of HOMO and LUMO discussed next.

#### Analysis of HOMO and LUMO

HOMO and LUMO are key concepts in molecular orbital theory, fundamental for understanding electronic transitions in materials science. HOMO is the highest energy orbital occupied by electrons and acts as an electron donor, where electrons can be excited or removed. LUMO is the lowest energy unoccupied orbital, functioning as an electron acceptor during excitation. Figure 3b, c shows the charge density distribution of HOMO and LUMO for the copper–carbon composite film material system when a copper atom is adsorbed at the bridge site, with and without an applied electric field.

Since the electron cloud is derived from the square of the normalized wave function, the charge density distribution also reflects the distribution of the wave function. Based on the charge density distribution, it can be observed that without an applied electric field, the wave function of the material as a whole exhibits an extended-state distribution. When an electric field is applied, the charge distribution around the copper atom appears more sparse compared to the case without the field, and the wave function is noticeably reduced. Similarly, the wave function distribution around the carbon atoms in the copper–carbon bond is also significantly reduced. Meanwhile, in other areas of the tube, the extended-state distribution of the wave function is markedly enhanced, and the charge density distributions of HOMO and LUMO appear staggered.

The enhancement of the extended state indicates that the copper–carbon composite film material system becomes more conductive under these conditions, as the wave function of the copper atom diffuses onto the nanocarbon film under the influence of the electric field, thereby increasing the number of electrons in the conductive system. At the same time, HOMO facilitates electron donation, while LUMO supports electron acceptance. The staggered distribution of HOMO and LUMO charge densities suggests that the occupied state distribution at this moment favors the formation of electron transport channels. This proves that the conductivity of the copper–carbon composite film material will be improved.

Expanding from a single copper atom to the entire copper–carbon composite film material system, when the nanocarbon film and copper film come into contact, theoretical calculations show that the copper–carbon distribution will be arranged in a bridge-site configuration, forming a network structure. In metallic crystals, electrons can move freely, allowing the copper film in the copper–carbon composite film material to provide a large number of transferable electrons. Meanwhile, the quasi-one-dimensional structure of the nanocarbon film enables ballistic electron transport. When an external electric field is applied, the staggered distribution of HOMO and LUMO charge densities will create channels more conducive to electron transport. Electrons from the numerous copper atoms in contact with the nanocarbon film migrate toward the carbon film layer, ultimately enhancing the conductivity of the copper–carbon composite film material.

#### Density of States Analysis

The density of states (DOS) describes the number of electronic states available at each energy level in a material, reflecting its electronic structure [51]. It determines how electrons populate energy levels under various conditions and is a critical concept in solid-state physics and materials science. DOS analysis evaluates this distribution, with the total DOS representing contributions from all orbitals and partial DOS focusing on specific orbitals [52]. It can be used to evaluate the distribution of electron states at different energy levels in copper–carbon composite film materials.

Figure 3d, e shows the total and partial DOS of the copper–carbon composite film material system before and after applying an external electric field. Without the electric field, the electronic properties of the copper–carbon system exhibit metallic characteristics. Below the Fermi level, the localized electronic states of copper atoms primarily consist of localized d-state electrons. After applying the electric field, significant changes appear in the DOS of the system. The DOS of the copper–carbon composite film system increases overall, with both occupied and unoccupied states showing a simultaneous increase. The total DOS crosses the Fermi level and extends into the conduction band. Additionally, the previously relatively localized s-state electrons nearly vanish, and the d-state electrons become more extended. This further verifies that the bonding between copper and carbon atoms has ionic characteristics. It is through bonding that the s-state electrons of copper atoms migrate to carbon atoms, creating a channel more favorable for electron transport and enhancing the conductivity of the composite material.

Based on the above calculations and mechanism analysis, it can be concluded that when the electrons of copper atoms migrate to the nanocarbon film under an applied electric field, a low-resistance electron transport network is formed. Due to the extremely high electron mobility of the nanocarbon film, this network greatly reduces scattering and resistance encountered by electrons during conduction, significantly enhancing the overall conductivity of the copper–carbon composite film.

### Large-Scale Production Equipment Based on this Study

**Fig. 4:**
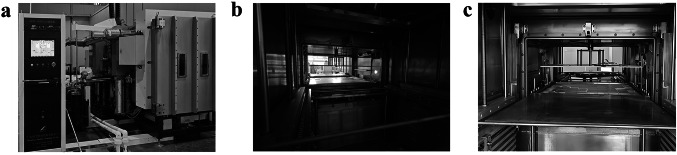
**a** Large-scale production equipment. **b** Internal structure of the deposition chamber. **c** RF electrode of large-scale production equipment

Based on the research results obtained from this study, a large-scale production apparatus was successfully developed in collaboration with partner companies, as shown in Fig. 4a–c. Figure 4a shows the large-scale deposition equipment along with its electronic control system. Figure 4b shows the internal structure of the deposition chamber. In the experiment, the diameter of the RF electrode plate used was 50 cm, whereas in the large-scale production apparatus, the RF electrode, as shown in Fig. 4c, reaches a diameter of 100 cm. This allows it to generate a larger cold plasma area, significantly enhancing the practical application value of carbon layer deposition technology under plasma immersion conditions.

Upgrading from laboratory-scale setups to industrial-scale production equipment enables plasma immersion thin film deposition technology and high-conductivity copper–carbon composite nanofilms to be widely applied in various high-performance electronic devices and electrical equipment that require copper conductivity to be enhanced.

## Conclusions

This study presents a novel copper–carbon nanofilm composite with significantly enhanced conductivity compared to copper nanofilms, revealing promising applications for electronic devices and electrical equipment. Copper possesses a high electron density but is limited by low electron mobility, which constrains its conductivity potential. To overcome this limitation, nanocarbon films with exceptional electron mobility were introduced, providing a significant advantage in enhancing the conductivity of copper nanofilms. Traditional carbon deposition methods, however, face limitations including deposition efficiency, interface quality, and scalability for industrial applications. By using plasma immersion carbon layer deposition technology, this study prepared multilayer copper–carbon nanofilms with superior interfacial electronic structures, achieving greater efficiency and better control during the preparation process. Experimental findings demonstrate that, for a five-layer copper–carbon nanofilm composite, conductivity markedly increases as the proportion of carbon nanofilm thickness increases. When the carbon nanofilm constitutes 16% of the total composite thickness, overall conductivity improves by 30.20% compared to pure copper.

The underlying mechanisms of this enhanced conductivity are studied through effective medium theory, first-principles calculations, and density of states analysis. The critical roles of copper atom adsorption sites and electron migration pathways within the nanocarbon layers were come up to explain this conductivity enhancement. Under an applied electric field, high-density electrons in the copper layer migrate into the nanocarbon film, establishing highly efficient electron transport channels and effectively reducing resistivity. Additionally, based on this research, we develop large-area electrode coating equipment that is highly suited for industrial production, providing a novel approach for enhancing conductivity and enabling the industrial application of copper–carbon nanocomposite films in the field of high-conductivity materials.

Certainly, challenges may still arise in practical production. For instance, maintaining consistency in plasma immersion technology during large-scale industrial applications and managing the associated equipment costs could pose significant constraints. Additionally, optimizing the structure and composition of the copper–carbon interface layer to ensure long-term stability under complex environmental conditions remains a critical area of research. Future studies could focus on developing more cost-effective large-scale thin-film deposition techniques to enhance the feasibility of industrial production, conducting in-depth investigations into the microstructural changes at the copper–carbon interface and their impact on electrical properties to provide theoretical guidance for material design, and assessing the performance of composite thin films under various environmental conditions while conducting application verifications to ensure reliability.
